# PKS5 Confers Cold Tolerance by Controlling Stomatal Movement and Regulating Cold-Responsive Genes in Arabidopsis

**DOI:** 10.3390/life12101633

**Published:** 2022-10-19

**Authors:** Chengyan Sun, Lin Zhu, Linlin Cao, Huimin Qi, Huijuan Liu, Fengyun Zhao, Xiuli Han

**Affiliations:** School of Life Sciences and Medicine, Shandong University of Technology, Zibo 255049, China

**Keywords:** cold stress, PKS5, stomatal aperture

## Abstract

Cold stress limits plant growth and development; however, the precise mechanisms underpinning plant acclimation to cold stress remain largely unknown. In this study, the Ser/Thr protein kinase SOS2-LIKE PROTEIN KINASE5 (PKS5) was shown to play a positive role in plant responses to cold stress. A PKS5 loss-of-function mutant (*pks5-1*) exhibited elevated sensitivity to cold stress, as well as a lower survival rate and increased ion leakage. Conversely, PKS5 gain-of-function mutants (*pks5-3*, *pks5-4*) were more tolerant to cold stress and exhibited higher survival rates and decreased ion leakage. Stomatal aperture analysis revealed that stomatal closure was slower during the first 25 min after cold exposure in *pks5-1* compared to wild-type, whereas *pks5-3* and *pks5-4* displayed accelerated stomatal closure over the same time period. Further stomatal aperture analysis under an abscisic acid (ABA) treatment showed slower closure in *pks5-1* and more rapid closure in *pks5-3* and *pks5-4.* Finally, expression levels of cold-responsive genes were regulated by PKS5 under cold stress conditions, while cold stress and ABA treatment can regulate *PKS5* expression. Taken together, these results suggest that PKS5 plays a positive role in short-term plant acclimation to cold stress by regulating stomatal aperture, possibly via ABA pathways, and in long-term acclimation by regulating cold-responsive genes.

## 1. Introduction

Cold stress is a major environmental factor restricting plant growth and development [1]. Cold stress decreases plant growth and yields by damaging cell structures and by inhibiting cell activities, for example, by damage to cell membranes and proteins by ice crystals, and by cold inhibition of photosynthesis [2]. Plants have evolved complex mechanisms to adapt to cold stress and improve their tolerance to freezing. The ICE1-CBF pathway plays a key role in cold stress responses in diverse plant species [1]. Upon exposure to cold stress, transcription factor ICE1 stimulates expression of *CBF* genes within 3 h. Within 24 h, CBFs activate the expression of cold-regulated (*COR*) genes that facilitate cold tolerance in plants [3,4]. Stomatal apertures are also thought to be involved in cold stress responses in plants [5,6]. Cold stress damage can be partially alleviated by H_2_S regulation of stomatal movement in concert with MPK4 [5,7]. At low soil temperatures (≤2 °C), photosynthesis rates and stomatal conductance were significantly reduced in high-elevation grasslands, indicating a role for stomatal movement in cold stress response [8]. Similarly, stomatal conductance decreased in Scots pine seedlings within 45 min of a cold stress treatment, whereas conductance started to increase with the extension of cold treatment time [6].

Abscisic acid (ABA) is an important plant hormone that regulates growth, development, and stress responses [9,10]. Under normal physiological conditions, ABA signaling is restricted by the inhibition of SnRK activity by clade A PP2Cs. Upon exposure to stress, ABA accumulates quickly and is recognized by its intracellular receptors (PYLs), then PYLs and PP2Cs form complexes to further release SnRK2 activity [11]. Protein kinases play roles in ABA-regulated stress responses. They include SnRK2.6, which is involved in regulating stomatal closure under osmotic and drought stresses [9], and Raf-like protein kinases, which regulate SnRK2 activity under osmotic stress [12]. ABA can regulate stomatal movements, and thereby photosynthetic rates, under various stress conditions, such as drought, salt, and cold exposure [13,14]. Although the mechanisms underlying ABA-regulated stomatal movements under osmotic and drought stresses have been well characterized [9], the regulatory mechanisms controlling stomatal movements under cold stress remain largely unknown.

SOS2-like protein kinase5 (PKS5) is a Ser/Thr protein kinase that plays an important role in the regulation of plant physiological activities. In the absence of salt stress, PKS5 negatively regulates plasma membrane (PM) H^+^-ATPase activity by preventing 14-3-3 protein binding to AHA2 [15]. PKS5 also inhibits SOS2 kinase activity by the promotion of 14-3-3 protein binding to SOS2 [16]. However, under salt stress, PKS5 interacts with J3 protein to release the activity of PM H^+^-ATPase, and PKS5 inhibition of SOS2 activity is released by the interaction between 14-3-3 and PKS5 [16,17]. PKS5 is also involved in ABA signal transduction via phosphorylation of abscisic acid-insensitive5 (ABI5), which regulates seed germination [18]. The stress-responsive roles of PKS5 in ABA signal transduction and PM H^+^-ATPase modulation suggest a role for PKS5 in stomatal movement regulation under stress conditions. PKS5 is thought to participate in stomatal movement regulation via formation of CBL5–PKS5 complexes and by stimulation of slow anion channel-associated1 (SLAC1) anion channel activity in stomatal guard cells [19]. Although changes in stomatal movement have been observed during cold stress [5], the underlying regulatory mechanisms remain unknown.

Whether PKS5 plays a role in plant cold stress response has not been reported. In this study, Arabidopsis PKS5 was found to act as a positive regulator during the response to cold stress. Seedlings of PKS5 loss-of-function mutants exhibited a cold-sensitive phenotype and slower stomatal closure under cold stress than wild-type plants. These results, together with the involvement of PKS5 in ABA signaling in previous studies and the similar pattern of stomatal movement between cold stress and ABA treatment in this study, suggest that PKS5 mediates plant cold responses, at least partially, via the regulation of ABA signal transduction.

## 2. Materials and Methods

### 2.1. Plant Materials and Growth Conditions

The following Arabidopsis strains were used in this study: *pks5-1* (SALK_108074) mutant and its wild-type Col-0, tilling mutants of *pks5-3* and *pks5-4* and their wild-type Col *erecta105* (BigM) [16].

Plants were grown on MS medium at 22 °C in a controlled environment growth chamber (YKNJ, Hefei youke, China) under a 16-h-light/8-h-dark photoperiod with the light intensity of 144 µmol·m^−2^·s^−1^. The growth chamber was equipped with LED light sources composed of red, blue, and far-red lights, with peak blue light at 460 nm and peak red light at 665 nm.

### 2.2. Freezing Tolerance Assay

The freezing tolerance assay was performed as described previously [3]. Arabidopsis seedlings were grown on MS medium containing 0.4% phytagel for two weeks at 22 °C. For the non-acclimated freezing tolerance treatment, plants were subjected to freezing at −5.5 °C for 5 h, then transferred to 4 °C under dark conditions for 12 h, and then shifted to normal growth condition of 22 °C with 16-h-light/8-h-dark photoperiod for the recovery of growth. For the cold-acclimated freezing tolerance treatment, plants were first pretreated at 4 °C for 3 d, then subjected to freezing at −9.5 °C for 6 h, then transferred to 4 °C under dark conditions for 12 h, and then shifted to normal growth conditions of 22 °C with 16-h-light/8-h-dark photoperiod for recovery of growth. Representative images were taken using a Nikon D5000 camera during the recovery growth at 22 °C under a 16-h-light/8-h-dark photoperiod.

For survival rate analysis, freezing-treated seedlings were recovered under normal growth condition of 22 °C with 16-h-light/8-h-dark photoperiod. The seedlings that could still grow new leaves were recorded as survivors, and the survival rates were calculated by the ratio of survived seedlings to total seedlings.

### 2.3. Ion Leakage Assay

The ion leakage assay was performed as previously described [3]. The freezing-treated seedlings were placed in a 15 mL tube containing 10 mL deionized water, whose electrical conductivity was detected and recorded as S0. The tube containing seedlings was vacuumed for about 5 min until the seedlings were totally immersed in the water, and then the tube was incubated for another 15 min on a shaking table at room temperature to obtain the electrical conductivity of S1. The tube was further boiled at 100 °C for 10 min, and the electrical conductivity was detected, which was recorded as S2. Finally, ion leakage was calculated by the formula: (S1 − S0)/(S2 − S0) × 100.

### 2.4. Stomatal Aperture Assay

The stomatal aperture assay was performed as previously described with minor changes [20]. Briefly, leaves of four-week-old seedlings were used for the assay. The abaxial epidermis was obtained by placing the abaxial surface of the leaf on a tape and removing mesophyll cells and adaxial epidermis quickly with another tape. Epidermal strips were then floated onto the opening buffer of 30 mM KCl, 10 mM MES-KOH, pH 6.15 for 1 h at 22 °C. For the stomatal aperture assay cold stressed leaves, strips were placed in the same opening buffer that had been pre-cooled at 4 °C for at least 1 h, and then investigated at the indicated times. For the stomatal aperture assay after ABA treatment, strips were moved from the opening buffer to the opening buffer with 100 µmol ABA, and then investigated at the indicated times. To maintain the accuracy of the experiments, the images of stoma at the indicated times were taken quickly, and then another ABA-treated strips at the indicated times were quickly put on the microscope and images were taken. Representative images were taken using a Nikon D5000 camera coupled to a Nikon Eclipse 55i microscope (magnification 20×). The stomatal aperture was measured using ImageJ.

### 2.5. qRT-PCR Analysis

Total RNAs were extracted from 14-d-old seedlings grown on MS medium using TRIzol reagent (Invitrogen). The extracted RNA was treated with RNase-free DNase I (Takara, Kusatsu, Japan) and reverse transcriptase (Promega, Madison, WI, USA) to remove genomic DNA and perform reverse transcription according to the manufacturer’s protocols. cDNA was then used for qPCR analysis. qPCR was performed on a 7500 real time PCR system (Life Technologies, USA) using the SYBR Premix Ex Taq Kit (Takara) according to the manufacturer’s protocol. Actin was used as an internal control and the relative expression levels of the detected genes were calculated as described previously [3]. Primers used in this study are listed in Table 1.

## 3. Results

### 3.1. PKS5 Is Essential for Plant Freezing Tolerance

**Figure 1:**
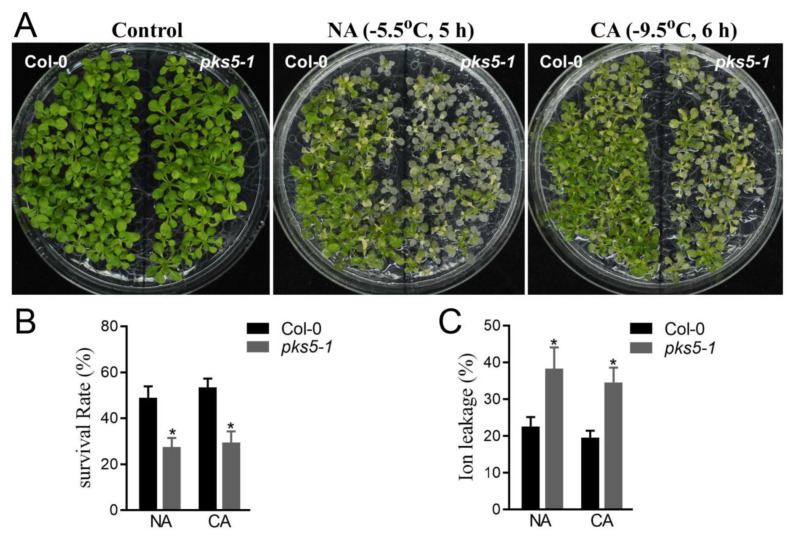
A deficiency of PKS5 impairs plant cold stress response. (**A**) Freezing phenotypes *pks5-1* under non−acclimated (NA) and cold−acclimated (CA) conditions. The wild−type Col−0 and *pks5-1* were grown on MS medium at 22 °C for 2 weeks before being subjected to the freezing treatment. For the NA treatment, seedlings were treated at −5.5 °C for 5 h; for the CA treatment, seedlings were pretreated at 4 °C for 3 d and then treated at −9.5 °C for 6 h. The freezing−treated seedlings were then transferred to 4 °C under a dark condition for 12 h, and then shifted to a normal growth condition at 22 °C for recovery of growth. Representative images were taken during the recovery growth at 22 °C. (**B**) Survival rates of *pks5-1* under NA and CA conditions. (**C**) Ion leakages of *pks5-1* under NA and CA conditions. Student’s *t*−test was used to analyze the statistical significance; each bar is the mean ± SD of three biological replications. Significant differences (*p* ≤ 0.05) in (**B**,**C**) are indicated by asterisks.

PKS5 has multiple regulatory roles in plant physiological processes. To identify whether PKS5 was involved in plant cold stress responses, a PKS5 loss-of-function mutant, *pks5-1*, was assessed in a freezing tolerance assay. As shown in Figure 1A, compared with wild-type Col-0 plants, *pks5-1* mutants exposed to a freezing treatment displayed elevated leaf withering under both non-acclimated and cold-acclimated conditions, indicating that PKS5 was essential for tolerance to cold stress.

Freezing treatment impairs cell structure and activity during cold exposure, and plants exposed to freezing exhibit decreased survival rates even after resumption of growth at normal temperatures. Compared with Col-0 seedlings, *pks5-1* mutant seedlings displayed a decreased survival rate after freezing under both non-acclimated and cold-acclimated conditions (Figure 1B).

Previous research reported that membrane damage caused by cold stress resulted in ion flow out of cells [3]. Ion leakage analysis of freeze-treated seedlings showed that *pks5-1* seedlings had a higher ion leakage rate than Col-0 plants under both non-acclimated and cold-acclimated conditions (Figure 1C). The cold-sensitive phenotype, decreased survival rate, and increased ion leakage exhibited by the *pks5-1* mutant under cold stress are indicative of an essential role for PKS5 in cold tolerance responses, with PKS5 playing a positive role in the response to cold stress.

### 3.2. Increases in PKS5 Activity Enhance Plant Freezing Tolerance

**Figure 2:**
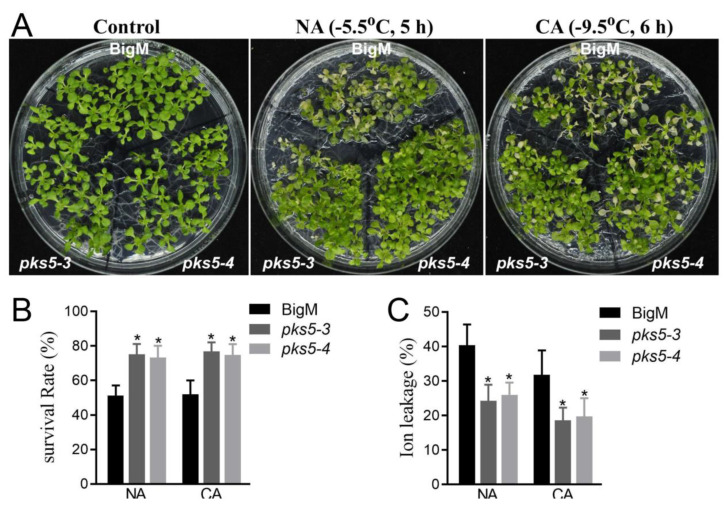
The increase of PKS5 activity improves plant cold stress response. (**A**) Freezing phenotypes *pks5-3* and *pks5-4* under NA and CA conditions. The wild−type BigM, *pks5-3* and *pks5-4* were grown on MS medium at 22 °C for 2 weeks before being subjected to freezing treatment. For the NA treatment, seedlings were treated at −5.5 °C for 5 h; for the CA treatment, seedlings were pretreated at 4 °C for 3 d and then treated at −9.5 °C for 6 h. The freezing−treated seedlings were then transferred to 4 °C under a dark condition for 12 h, and then shifted to a normal growth condition at 22 °C for recovery of growth. Representative images were taken during the recovery growth at 22 °C. (**B**) Survival rates of *pks5-3* and *pks5-4* under NA and CA conditions. (**C**) Ion leakage of *pks5-3* and *pks5-4* under NA and CA conditions. Student’s t−test was used to analyze the statistical significance; each bar is the mean ± SD of three biological replications. Significant differences (*p* ≤ 0.05) in (**B**,**C**) are indicated by asterisks.

The regulatory role of PKS5 in the response to cold stress was further assessed using two previously developed PKS5 gain-of-function mutants, *pks5-3* and *pks5-4*, which have elevated PKS5 activity levels [17]. Seedlings of *pks5-3*, *pks5-4*, and the corresponding wild-type (BigM), were exposed to freezing treatment. Compared with BigM, *pks5-3* and *pks5-4* displayed a freezing-tolerant phenotype under both non-acclimated and cold-acclimated conditions (Figure 2A). This result indicates that increasing PKS5 activity can improve plant freezing tolerance.

Analysis of seedling survival after freezing treatment showed that *pks5-3* and *pks5-4* displayed higher survival rates than BigM under both non-acclimated and cold-acclimated conditions (Figure 2B). The ion leakage assay of the freeze-treated seedlings also showed that, compared with BigM, *pks5-3* and *pks5-4* displayed lower ion leakage after freezing treatment (Figure 2C).

These results indicate that increasing PKS5 activity enhances plant freezing tolerance and suggest that PKS5 positively regulates freezing tolerance in Arabidopsis.

### 3.3. PKS5 Regulates Stomatal Movements under Cold Stress

**Figure 3:**
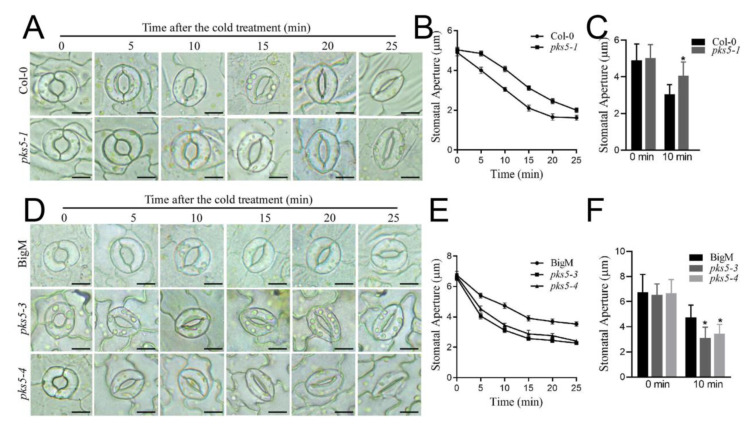
PKS5 regulates stomatal movements under cold stress. (**A**) Representative stomatal aperture of Col−0 and *pks5-1* under cold treatment at 0, 5, 10, 15, 20 and 25 min. Abaxial epidermal strips were cultured in the opening buffer in the light for 1 h to open the stomata. Strips were then treated at 4 °C and investigated at the indicated times. Scale bar = 10 µm. (**B**) Stomatal apertures measured from (**A**). (**C**) Statistical analysis of stomatal aperture of Col−0 and *pks5-1* after 10 min cold treatment from (**A**). (**D**) Representative stomatal aperture of BigM, *pks5-3*, and *pks5-4* under cold treatment at 0, 5, 10, 15, 20 and 25 min. Abaxial epidermal strips were cultured in the opening buffer in the light for 1 h to open the stomata. Strips were then treated at 4 °C and investigated at the indicated times. Scale bar = 10 µm; (**E**) Stomatal apertures measured from (**D**). (**F**) Statistical analysis of stomatal aperture of BigM, *pks5-3*, and *pks5-4* after 10 min cold treatment from D. Student’s t−test was used to analyze the statistical significance; each bar is the mean ± SD of three biological replications (*n* > 50). Significant differences (*p* ≤ 0.05) in (**C**,**F**) are indicated by asterisks.

Photosynthesis and stomatal movement are regulated during plant responses to cold stress [8]. To investigate whether PKS5 can regulate plant cold stress responses via the regulation of stomatal movement, stomatal aperture in response to cold stress was assessed in loss-of-function *pks5-1* seedlings. Stomata in both Col-0 and loss-of-function *pks5-1* seedlings were closed in 25 min after initiation of cold exposure; however, *pks5-1* stomata closed more slowly than those in Col-0, with a significant difference in stomatal closure observed between Col-0 and *pks5-1* at 10 min after cold initiation (Figure 3A–C). This result indicates that loss of PKS5 function impairs the regulation of stomatal movement during exposure to cold stress.

The role of PKS5 in stomatal movement was assessed further using *pks5-3* and *pks5-4* gain-of-function mutants. Stomata in *pks5-3*, *pks5-4*, and BigM were closed in 25 min after cold treatment initiation (Figure 3D). However, closure of stomata occurred more quickly in *pks5-3* and *pks5-4* than in BigM, and a significant difference in stomatal closure was observed between BigM and *pks5-3* and *pks5-4* at 10 min after cold initiation (Figure 3E,F). This suggests that increasing PKS5 activity increases the rate of stomatal closure under cold stress.

Together, these observations indicate that PKS5 plays a regulatory role in stomatal movements during plant cold stress responses.

### 3.4. PKS5 Mediates ABA-Regulated Stomatal Movements

**Figure 4:**
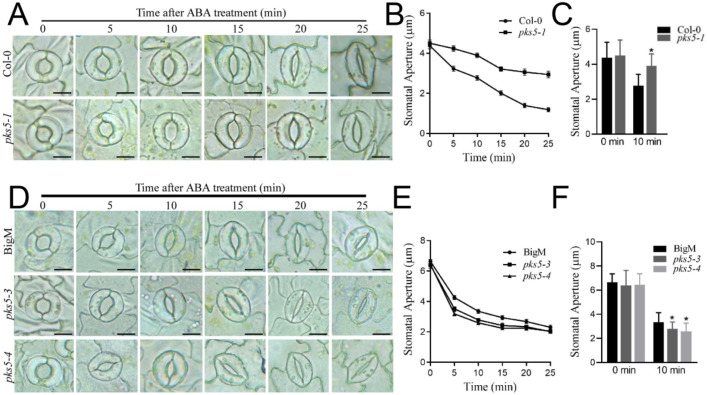
PKS5 mediates ABA−regulated stomatal movements. (**A**) Representative stomatal aperture of Col−0 and *pks5-1* under ABA treatment at 0, 5, 10, 15, 20 and 25 min. Abaxial epidermal strips were cultured in the opening buffer in the light for 1 h to open the stomata. Strips were then treated with ABA−containing buffer and investigated at the indicated times. Scale bar = 10 µm. (**B**) Stomatal apertures measured from (**A**). (**C**) Statistical analysis of stomatal aperture of Col−0 and *pks5-1* after 10 min ABA treatment from (**A**). (**D**) Representative stomatal aperture of BigM, *pks5-3*, and *pks5-4* under ABA treatment at 0, 5, 10, 15, 20 and 25 min. Abaxial epidermal strips were cultured in the opening buffer in the light for 1 h to open the stomata. Strips were then treated with ABA−containing buffer and investigated at the indicated times. Scale bar = 10 µm. (**E**) Stomatal apertures measured from (**D)**. (**F**) Statistical analysis of stomatal aperture of BigM, *pks5-3*, and *pks5-4* after 10 min ABA treatment from (**D**). Student’s t−test was used to analyze the statistical significance; each bar is the mean ± SD of three biological replications (*n* > 50). Significant differences (*p* ≤ 0.05) in (**C**,**F**) are indicated by asterisks.

ABA levels were previously shown to increase upon exposure to cold stress, and ABA is known to play a role in the regulation of stomatal movement [13,21,22]. To investigate whether the PKS5-regulated stomatal movement observed after cold exposure was related to ABA signaling, stomatal apertures were examined after ABA treatment in the PKS5 loss-of-function mutant, *pks5-1*, and gain-of-function mutants *pks5-3* and *pks5-4*. ABA treatment induced stomatal closure in both Col-0 and *pks5-1*, but *pks5-1* stomata closed more slowly than those in Col-0 (Figure 4A,B). A significant difference in stomatal closure was observed between Col-0 and *pks5-1* at 10 min after treatment (Figure 4C). ABA treatment also induced stomatal closure in *pks5-3*, *pks5-4*, and BigM, and *pks5-3* and *pks5-4* stomata closed more quickly than those in BigM (Figure 4D,E). A significant difference in stomatal closure was observed between BigM and the *pks5-3* and *pks5-4* mutants at 10 min after treatment (Figure 4F). These results suggest that the regulatory role of PKS5 in stomatal movement is related to ABA signaling, and that cold-induced ABA accumulation may contribute to the regulation of stomatal movement by PKS5 under cold stress.

### 3.5. Cold-Responsive Genes Regulated by PKS5 under Cold Stress

**Figure 5:**
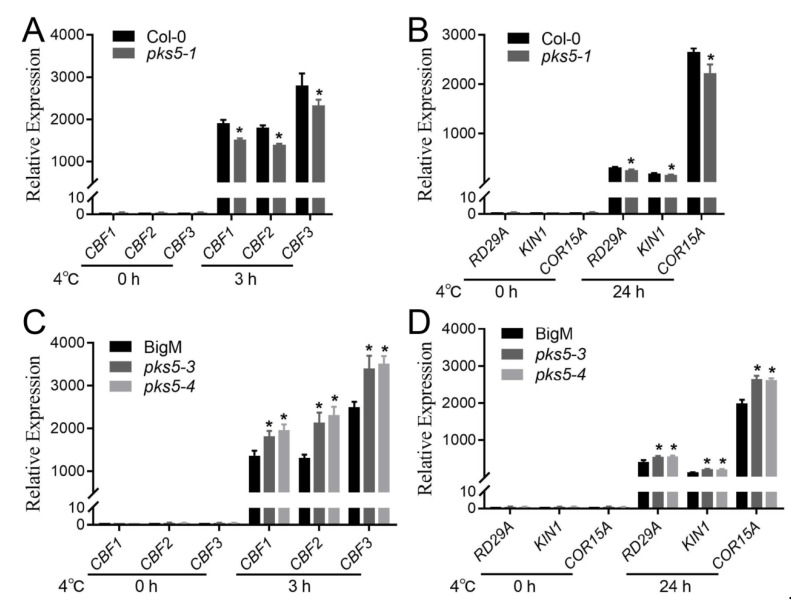
PKS5 regulates cold−responsive genes under cold stress. (**A**) Expression level analysis of *CBF* genes in *pks5-1*. (**B**) Expression level analysis of CBF target genes in *pks5-1*. (**C**) Expression level analysis of *CBF* genes in *pks5-3* and *pks5-4*. (**D**) Expression level analysis of CBF target genes in *pks5-3* and *pks5-4*. Seedlings grown for 2 weeks on MS medium at 22 °C were treated at 4 °C for 3 h (**A**,**C**) to analyze *CBF* gene expression and were treated at 4 °C for 24 h to analyze CBF target gene expression. Gene expression levels in untreated wild−type seedlings (Col−0 in A and B, BigM in (**C**,**D**)) were set to 1. Student’s t−test was used to analyze the statistical significance; each bar is the mean ± SD of three biological replications. Significant differences (*p* ≤ 0.05) in (**A**–**D**) are indicated by asterisks.

Cold-responsive genes, such as *CBF* and *COR*, are upregulated upon exposure to cold stress in plants [1]. To explore whether *CBF* and *COR* genes were involved in the PKS5-regulated cold stress response, expression levels of cold-responsive genes, including *CBF1*, *CBF2*, *CBF3*, *COR15A*, *KIN1*, and *RD29A*, were examined in wild-type, *pks5-1*, *pks5-3*, and *pks5-4* plants. At 22 °C, gene expression was comparable between *pks5-1* and Col-0, and between *pks5-3*, *pks5-4*, and BigM (Figure 5A–D). However, cold-induced expression of *CBF* genes was lower in *pks5-1*, and higher in *pks5-3* and *pks5-4*, compared to their respective wild-types (Figure 5A,C). Moreover, three CBF-regulated genes, *COR15A*, *KIN1*, and *RD29A*, also exhibited lower expression in *pks5-1*, and higher expression in *pks5-3* and *pks5-4*, compared to their respective wild-types (Figure 5B,D). These results suggest that PKS5 has a positive regulatory role in acclimation of plants to cold stress, and that this regulation is mediated, at least partially, via regulation of expression of *CBF* and CBF-regulated genes.

### 3.6. Cold Stress and ABA Treatment Regulate PKS5 Expression

**Figure 6:**
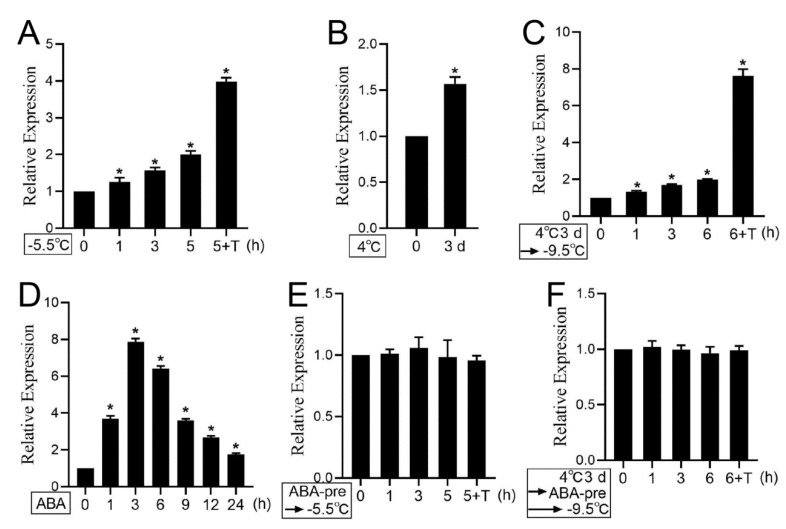
Expression level analysis of *PKS5* under cold stress and ABA treatment. (**A**) Expression level analysis of *PKS5* under cold stress in non−acclimated seedlings. (**B**) Expression level analysis of *PKS5* after cold acclimation. (**C**) Expression level analysis of *PKS5* under cold stress in cold−acclimated seedlings. (**D**) The expression level analysis of *PKS5* under ABA treatment. (**E**) ABA effect on *PKS5* expression under freezing stress in non−acclimated seedlings. (**F**) ABA effect on *PKS5* expression under freezing stress in cold−acclimated seedlings. The two−week old Col−0 seedlings grown on MS medium at 22 °C were subjected to various treatments. For freezing treatment in non−acclimated seedlings, seedlings were frozen at −5.5 °C and collected for *PKS5* expression level analysis. For the freezing treatment in cold−acclimated seedlings, seedlings underwent a cold acclimation process of 4 °C for 3 d, and were then subjected to freezing at −9.5 °C. For the ABA treatment, seedlings were first sprayed with ddH_2_O containing 10 µM ABA, and then the seedlings were collected for *PKS5* expression. For the ABA−pretreatment, seedlings were pretreated with 10 µM ABA for 3 h, and then non−acclimated seedlings and cold−acclimated seedlings were subjected to freezing. T: transition period at 4 °C under dark for 12 h. Actin was used as an internal control. Gene expression levels in wild−type Col−0 seedlings at 0 h were set to 1. Student’s *t*−test was used to analyze the statistical significance; each bar is the mean ± SD of three biological replications. Significant differences (*p* ≤ 0.05) in (**A**–**D**) are indicated by asterisks.

To further explore whether the expression of *PKS5* was induced by cold stress, expression levels of *PKS5* were analyzed under freezing treatment. The results showed that a freezing treatment of −5.5 °C induced elevated expression of *PKS5*, especially during the transition period at 4 °C overnight (Figure 6A). In this study and previous studies, cold acclimation at 4 °C for 3 d could significantly improve plant freezing tolerance [3]. Consistent with the improved freezing phenotype, *PKS5* expression level could also be induced during the cold acclimation process (Figure 6B). Freezing treatment on cold-acclimated seedlings also showed that *PKS5* expression could be induced by the freezing treatment, especially during the transition period at 4°C overnight (Figure 6C). However, the freezing-treated, cold-acclimated seedlings showed a substantially greater increase of *PKS5* expression level (about 8-fold) compared with that in non-acclimated seedlings (about 4-fold) during the transition period at 4 °C overnight (Figure 6A,C). These results indicate that the *PKS5* gene can be induced by cold stress.

To further investigate ABA effect on *PKS5* expression, *PKS5* expression was first examined by ABA treatment under normal temperature. The result showed that *PKS5* expression could be induced by ABA treatment, with the highest induced level at 3 h (Figure 6D). To investigate the effect of cold-induced ABA accumulation on PKS5 expression, seedlings of non-acclimated and cold-acclimated were both pretreated with 10 µM ABA for 3 h, and then treated with freezing stress; however, no increase in *PKS5* expression was observed in both non-acclimated seedlings and cold-acclimated seedlings (Figure 6E,F). These results suggest that cold-induced PKS5 expression might be regulated by ABA accumulated under cold stress.

## 4. Discussion

Exposure of plants to cold stress stimulates a cascade of short-term and long-term physiological activities that mitigate cellular damage. Regulation of gene expression in response to cold exposure is well understood, and includes *CBF* genes, which are upregulated within 1–3 h of exposure, and *COR* genes, which are upregulated later, 12–24 h after cold exposure [3]. Previous research showed that cold stress induced stomatal movement. Stomatal conductance in Scots pine seedlings first decreased within 45 min of the freezing period of freeze-thaw treatment, then subsequently increased with the extension of freezing treatment time [6]. In this study, the PKS5 loss-of-function mutant *pks5-1*, which exhibited a cold-sensitive phenotype, exhibited slower stomatal closure during the first 25 min after cold exposure than wild-type seedlings, and also exhibited lower expression of *CBF* and *COR* genes 3 h and 24 h after cold exposure, respectively. Conversely, PKS5 gain-of-function mutants *pks5-3* and *pks5-4*, which were cold-tolerant, exhibited faster stomatal closure during the first 25 min after cold exposure than wild-type, and also exhibited higher expression of *CBF* and *COR* genes 3 h and 24 h after cold exposure, respectively. Stomatal aperture regulation was examined at 10 min intervals from 25 min to 3 h after cold exposure, but no clear regulatory pattern was apparent (data not shown). These observations in Arabidopsis are similar to previous research results examining stomatal closure in Scots pine seedlings, which also responded to cold stress by regulating stomatal closure shortly after cold exposure [6]. This study confirms the involvement of PKS5 in stomatal aperture regulation under cold stress in Arabidopsis; however, the detailed regulatory mechanisms require further elucidation.

Cold stress can be divided into chilling stress (0–15 °C) and freezing stress (<0 °C) [1]. Since freezing stress leads to ice formation, and is more harmful to the plant, plants have evolved cold acclimation to response to freezing stress. *CBFs* expression is rapidly upregulated under chilling stress at 4 °C, which further activates downstream *COR* genes, leading to an increase of freezing tolerance via the biosynthesis of osmolytes, such as soluble sugars [1,21]. It is reported that 14-3-3 proteins negatively regulate freezing tolerance [23]. Under cold stress, plasma membrane kinase CRPK1 phosphorylates 14-3-3 proteins, which are then translocated from cytosol into nucleus to facilitate the degradation of CBF proteins [23]. It is interesting that PKS5 can interact with 14-3-3 proteins under salt stress, and 14-3-3 proteins can bind to salt-induced Ca^2+^ and repress PKS5 activity. Although the CBF cold signaling pathway has been extensively studied, the upstream components regulated by calcium signaling remain largely unknown. Whether PKS5-regulated cold stress response involves calcium signaling needs further study.

PKS5 was previously shown to have a role in ABA signal transduction, and ABA treatment in this study resulted in similar stomatal closure patterns to those seen after cold treatment. However, the downstream or upstream elements involved in PKS5-regualted stomatal movement, and the mechanism by which PKS5 mediates ABA-regulated stomatal movements, require further investigation. Under normal cultivation conditions, PKS5 can interact with and phosphorylate SOS2 at Ser-294, inhibiting SOS2 activity [16]. PKS5 can also phosphorylate PM H^+^-ATPase at Ser-931 to inhibit PM H^+^-ATPase activity [15]. Conversely, upon exposure to salt stress, PKS5 can interact with J3 and 14-3-3 to activate PM H^+^-ATPase and SOS2 [16,17]. During ABA-regulated seed germination, PKS5 can interact with ABI5 and phosphorylate ABI5 at Ser-42 to play a positive role in ABA signaling [18]. PKS5 also participates in plant defense responses via phosphorylation of NPR1 (Nonexpressor of Pathogenesis-Related gene 1) at the C-terminal region [24]. The guard cell anion channel SLAC1 has a fundamental role in the regulation of stomatal aperture control, and PKS5 can form CBL5–CIPK11 complexes to stimulate SLAC1 activity [19]. These PKS5-interacting proteins may participate in PKS5-regulated stomatal aperture closure under cold stress conditions.

**Figure 7:**
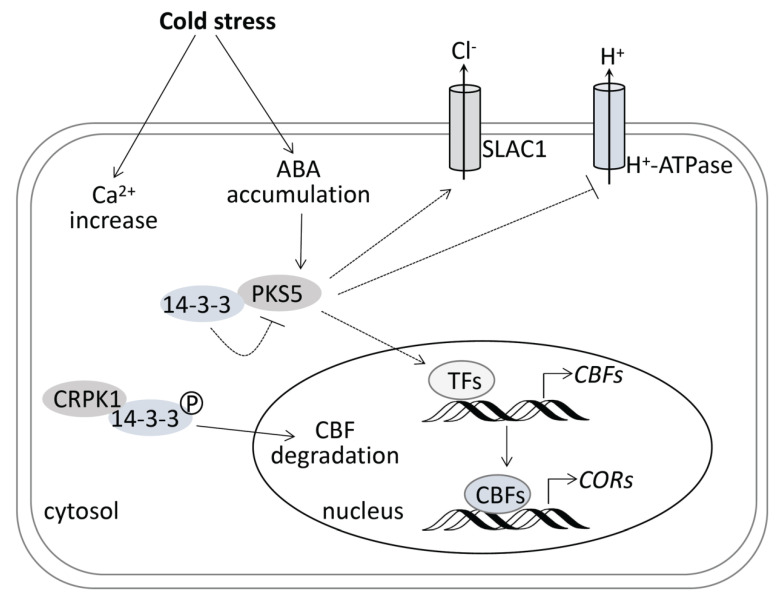
Model for PKS5 in regulating freezing tolerance in Arabidopsis. Cold stress induces rapid increase of cytosolic calcium (Ca^2+^) and the accumulation of ABA. Cold−induced ABA accumulation, on the one hand, induces *PKS5* expression under cold stress and, on the other hand, regulates PKS5 role in the regulation of stomatal movement under cold stress. PKS5 may stimulate SLAC1 anion channel activity through the formation of CBL5−PKS5 complex, or directly inhibit PM H^+^−ATPase activity by the phosphorylation of AHA2 at Ser−931 to regulate stomatal movement under cold stress in short−term plant acclimation to cold stress. In long−term plant acclimation to cold stress, PKS5 can indirectly upregulate the expression of *CBFs* and *CORs* genes through unknown transcriptional factors (TFs) to increase the osmolyte synthesis to improve plant freezing tolerance. The 14−3−3 proteins play negative roles in plant response to cold stress, which, on the one hand, inhibit PKS5 activity through their interaction and, on the other hand, can be phosphorylated by CRPK1 and translocated into nucleus, leading to CBF degradation. Broken arrows indicate activation unconfirmed to occur under cold stress.

Cold stress induces a rapid increase of cytosolic calcium (Ca^2+^) and the accumulation of ABA. Whether 14-3-3 proteins can bind to cold-induced Ca^2+^ and regulate PKS5 activity may help decode Ca^2+^ signaling in the cold stress response. SLAC1 is required for plant guard cell S-type anion channel function in stomatal signaling [25], and PKS5 can regulate SLAC1 anion channel activity through formation of the CBL5-PKS5 complex [19]. Whether SLAC1 plays a role in the cold stress response, and whether PKS5 involves this process, are still unknown and need further study. PM H^+^-ATPase in plant is an essential enzyme with multiple physiological functions, and is highly regulated, mainly by phosphorylation [26]. PM H^+^-ATPase regulates stomatal movements under various conditions, such as jasmonate-regulated stomatal closure [27], light-induced stomatal opening [28], and salt stress response [17]. Although PM H^+^-ATPase has been reported to be involved in cold stress responses [29], there is still no direct evidence about how it regulates cold stress responses. PKS5 might be a link between PM H^+^-ATPase and cold stress response. Whether PKS5 plays a role mediating Ca^2+^ signaling in the cold stress response, and which transcriptional factors (TFs) mediate its regulation of *CBFs* and *CORs* genes, needs further study. The model depicted in Figure 7, as a combination of this study and previous studies, may help explain the mechanism of plant responses to cold stress.

## Figures and Tables

**Table 1:** Primers for RT-PCR in this study.

Primer	Sequence (5′-3′)
*PKS5*-F	GAAGGTGCTAAAGTTGATGTATGGTCT
*PKS5*-R	CGTCATCGTGGAACTTGATCTGTTT
*CBF1*-F	GCATGTCTCAACTTCGCTGA
*CBF1*-R	ATCGTCTCCTCCATGTCCAG
*CBF2*-F	TGACGTGTCCTTATGGAGCTA
*CBF2*-R	CTGCACTCAAAAACATTTGCA
*CBF3*-F	GATGACGACGTATCGTTATGGA
*CBF3*-R	TACACTCGTTTCTCAGTTTTACAAAC
*COR15A*-F	GCTTCAGATTTCGTGACGGATAAAAC
*COR15A*-R	GCAAAACATTAAAGAATGTGACGGTG
*KIN1*-F	ACCAACAAGAATGCCTTCCA
*KIN1*-R	CCGCATCCGATACACTCTTT
*RD29A*-F	GCCGAGAAACTTCAGATTGG
*RD29A*-R	CCATTCCTCCTCCTCCTTTC
*ACTIN2/8*-F	GGTAACATTGTGCTCAGTGGTGG
*ACTIN2/8*-R	AACGACCTTAATCTTCATGCTGC

## Data Availability

Not applicable.
